# Stigmasterol Decreases Oncostatin M Production Through Suppressing PI3K/Akt/NF-κB Signaling Processes in Neutrophil-like Differentiated HL-60 Cells

**DOI:** 10.3390/biomedicines14010220

**Published:** 2026-01-20

**Authors:** Na-Ra Han, Hi-Joon Park, Seong-Gyu Ko, Phil-Dong Moon

**Affiliations:** 1 College of Korean Medicine, Kyung Hee University, Seoul 02447, Republic of Korea; nrhan@khu.ac.kr; 2 Korean Medicine-Based Drug Repositioning Cancer Research Center, College of Korean Medicine, Kyung Hee University, Seoul 02447, Republic of Korea; epiko@khu.ac.kr; 3 Department of Anatomy & Information Sciences, College of Korean Medicine, Kyung Hee University, Seoul 02447, Republic of Korea; acufind@khu.ac.kr; 4 Department of Preventive Medicine, College of Korean Medicine, Kyung Hee University, Seoul 02447, Republic of Korea; 5 Center for Converging Humanities, Kyung Hee University, Seoul 02447, Republic of Korea

**Keywords:** oncostatin M, stigmasterol, dHL-60 cells, GM-CSF, neutrophils, phosphatidylinositol 3-kinase, Akt, nuclear factor-κB

## Abstract

**Background**: Cytokine oncostatin M (OSM) is implicated in inflammatory conditions. The plant sterol stigmasterol (ST) is found in diverse plant foods and exerts various benefits, such as antitumor, antioxidant, and anti-inflammatory effects. However, the inhibitory mechanism of ST on OSM production in neutrophils needs to be elucidated. **Methods**: To evaluate the modulatory effects of ST, this investigation employed neutrophil-like differentiated (d)HL-60 cells. ELISA, real-time PCR, Western blotting, and immunofluorescence staining were conducted. dHL-60 cells were pretreated with ST (0.02 to 2 µg/mL) for 1 h, and then stimulated with GM-CSF (5 ng/mL). **Results**: Our results showed that addition of granulocyte–macrophage colony-stimulating factor (GM-CSF) leads to up-regulation of OSM mRNA and protein in dHL-60 cells, while pretreatment with ST reduces OSM mRNA and protein levels. Mechanistically, the highest dose (2 µg/mL) of ST significantly decreased phosphorylation of phosphatidylinositol 3-kinase, protein kinase B (Akt), and nuclear factor-κB. **Conclusions**: Our findings suggest that the plant sterol ST shows potential and warrants in vivo validation on OSM regulation via suppressing PI3K/Akt/NF-κB Signaling Processes.

## 1. Introduction

It was initially reported that OSM treatment induces inhibition of proliferation of cancer cells [1]. Numerous reports have focused on cancer-related research and suggested that OSM up-regulation was found in patients with tumor as well as cancer tissues [2,3,4]. Several studies, meanwhile, suggested that OSM is involved in inflammation reactions and is secreted in diverse cells, including monocytes, activated T cells, dendritic cells, macrophages, and neutrophils [5,6,7,8,9].

Numerous studies have suggested that various pathologic conditions, inclusive of cancer development, the formation of blood cells, reorganization of the extracellular matrix, the formation of hepatic tissues, heart reconstruction, and inflammation, are related to OSM [2,10,11,12,13]. Among them, inflammation is an essential part where OSM performs a wide range of activities [2]. Recent studies [2,14] have reported that OSM plays an important role in inflammation in arthritic and hepatic disorders. Furthermore, some studies suggested that OSM is implicated with inflammatory respiratory diseases [15,16], in addition to inflammatory reactions being increased by exposure to human OSM protein in intestinal stromal cells [5]. Liu et al. [9] presented that exposure to OSM protein leads to elevated inflammatory reactions in keratinocyte cell line HaCaT cells. Our previous work also presented that stimulation with OSM protein results in increased IL-1β levels in HaCaT cells, implying that OSM is involved in inflammatory responses [17]. It has been reported that main source of OSM is neutrophils in respiratory disorders [16]. Generally, many studies have employed HL-60 to explore functions of neutrophil cells, because primary neutrophils have a short life span and variabilities in donors [17]. Treatment with dimethyl sulfoxide (DMSO) led to neutrophil-like differentiated cells in HL-60 cells [18,19]. Neutrophil-like differentiated (d)HL-60 cells have been employed in lots of studies to investigate the functions of neutrophils [18,20]. We hypothesized that stigmasterol (ST) reduces GM-CSF-induced OSM production in neutrophils via inhibition of PI3K/Akt/NF-κB signaling. Even though dHL-60 cells were used in this study, dHL-60 cells are a surrogate and do not fully recapitulate primary neutrophil complexity. However, to control for donor variability while investigating a specific signaling pathway, dHL-60 cells were used in this study. Neutrophils are important cells that arrive at sites of inflammation. Their recruitment requires them to migrate through the endothelial cell monolayer. Endothelium-derived GM-CSF influences expression of OSM during inflammation [21]; thus, GM-CSF stimulation was used in the present study.

Accumulating evidence indicates that dysregulation of the PI3K/Akt and NF-κB signaling cascades plays a central role in controlling inflammatory responses, cell survival, and cytokine production across diverse pathological conditions. Pharmacological modulation of PI3K/Akt signaling has been shown to suppress aberrant cellular behavior and inflammatory mediator expression in cancer and inflammatory disease models, highlighting its relevance as a therapeutic target [22]. Similarly, NF-κB acts as a master transcriptional regulator of inflammation, and its inhibition effectively attenuates pathological cell activation and proliferation [23,24]. Upstream activation of NF-κB through pathways such as TLR4 further contributes to sustained inflammatory signaling in diseases including inflammatory bowel disease and neuroinflammation [25,26]. Recent systems-level studies integrating network pharmacology and transcriptomic analyses demonstrate that bioactive natural compounds often exert anti-inflammatory effects through coordinated modulation of multiple signaling pathways rather than single molecular targets [27,28]. In parallel, clinical and experimental evidence underscores the pathogenic importance of neutrophils in inflammatory disorders, with elevated neutrophil-associated biomarkers correlating with disease risk and severity [29].

The plant sterol ST is found in diverse plant foods and exerts various benefits, such as antitumor, antioxidant, and anti-inflammatory effects [30]. Regarding inflammatory diseases, it has been suggested that ST inhibits airway hyperresponsiveness and airway inflammation in asthmatic mice [31]. Furthermore, ST alleviated chronic obstructive pulmonary disease through down-regulation of PI3K and Akt phosphorylation in lung tissues of rat [32]. In this study, we thus examined whether ST regulates OSM release through the PI3K/Akt/NF-κB signal pathway in dHL-60 cells.

## 2. Materials and Methods

### 2.1. Preparation of ST

ST (Sigma-Aldrich Co., Ltd., St. Louis, MO, USA) was prepared by dissolving it in the minimum amount of pure ethanol, following established protocols [33,34], and the doses were determined based on previous research [30,35,36]. Dilutions were made in phosphate-buffered saline (PBS) and filtered through 0.22-μm syringe filter. Maximal final concentration of ethanol was 0.04% (*v*/*v*).

### 2.2. Cell Culture

**Figure 1:**
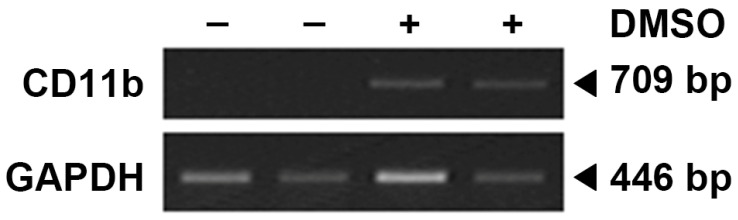
Neutrophil marker in HL-60 cells (DMSO: −) and differentiated HL-60 cells (DMSO: +). CD11b is known to be expressed in neutrophil-differentiated cells but not in undifferentiated ones.

The HL-60 cell line was obtained from Korean Cell Line Bank (Seoul, Republic of Korea) and maintained in RPMI 1640 (Gibco, Grand Island, NY, USA) with 10% FBS, 37 °C, and 5% CO_2_. Freezing medium (RPMI1640, 52.5%; FBS, 40%; DMSO, 7.5%) was used for cryopreservation. To obtain neutrophil-like dHL-60 cells, HL-60 cells were differentiated in the presence of 1.3% dimethyl sulfoxide (DMSO) over 7 days with a medium change (fresh RPMI 1640 medium containing 1.3% DMSO on 4th day) as described previously [17,21]. The neutrophil marker (CD11b) was checked (Figure 1).

### 2.3. MTT Assay

Cytotoxicity was assessed by means of MTT assay as described previously [37,38]. dHL-60 cells (5 × 10^4^ cells in 500 μL of medium) were seeded in a 24-well plate and pretreated with ST or vehicle (PBS containing 0.04% ethanol) for 1 h, and then stimulated with GM-CSF (5 ng/mL, Cat. No. 295-OM, R&D system Inc., Minneapolis, MN, USA) for 4 h. The cells were incubated with 3-(4,5-dimethylthiazol-2-yl)-2,5-diphenyltetrazolium bromide (MTT, 500 μg/mL, Sigma-Aldrich Co., Ltd., St. Louis, MO, USA) solution at 37 °C for 4 h. Next, we added 1 mL of DMSO to dissolve the MTT formazan, and transferred 100 μL of supernatant into a new 96-well microplate. A microplate reader (540 nm, Versa Max, Molecular Devices, Sunnyvale, CA, USA) was used to measure the absorbance of formazan dissolved in DMSO.

### 2.4. Enzyme-Linked Immunosorbent Assay (ELISA)

OSM production reached the maximum level 4 h after exposure to GM-CSF, as determined in our previous study [17]. dHL-60 cells (2.5 × 10^5^ cells in 500 μL of medium) were seeded in a 24-well plate and pretreated with ST or vehicle for 1 h, and then stimulated with GM-CSF (5 ng/mL) for 4 h. OSM levels were assessed by the ELISA method as previously described [39,40,41,42]. The capture antibody (Cat. No. MAB295, 4 μg/mL, R&D system Inc., Minneapolis, MN, USA) was pre-coated in a 96-well plate overnight. PBS containing 10% FBS was added to block the plate for 2 h. After washing the plate with PBS containing Tween 20 (PBST), cell supernatants were added into the plate for 2 h. After washing the plate with PBST, the plate was treated with biotinylated detection antibody (Cat. No. BAF295, 0.2 μg/mL, R&D system Inc., Minneapolis, MN, USA) for 2 h and then incubated with avidin conjugated to horseradish peroxidase (Sigma-Aldrich Co., Ltd., St. Louis, MO, USA) for 30 min. Absorbance by TMB substrate (BD Pharmingen, San Jose, CA, USA) was measured by a microplate reader (405 nm, Versa Max, Molecular Devices, Sunnyvale, CA, USA). The inhibition percentage of OSM release was calculated using the following equation:

% inhibition = {(C − B) − (S − B)} × 100/(C − B)

where B is OSM release (vehicle + PBS), C is the OSM release (vehicle + GM-SCF), and S is the OSM release (ST + GM-CSF).

### 2.5. qRT-PCR

Exposure to GM-CSF led to the maximum mRNA expression of OSM in 30 min as determined in our previous study [17]. dHL-60 cells (2 × 10^6^ cells in 2 mL of medium) were seeded in 6-well plate and pretreated with ST or vehicle for 1 h, and then stimulated with GM-CSF (5 ng/mL) for 30 min. The harvested cells were used to isolate total RNA by means of an RNA extraction reagent (iNtRON Biotech, Seongnam, Republic of Korea) as previously described [43,44]. Total RNA concentrations and purity ratios (260/280 and 260/230) were measured using a NanoDrop 2000 UV–vis Spectrophotometer (Thermo Fisher Scientific, Wilmington, DE, USA). Total RNA (2.0 μg) was heated at 70 °C for 5 min and then chilled on ice. The first-strand cDNA from total RNA was synthesized with cDNA synthesis reagents (Bioneer, Daejeon, Republic of Korea) at 42 °C for 60 min. The following designed primers were used for the real time PCR (Applied Bio-systems, Foster City, CA, USA) by using Power SYBR^®^ Green Master Mix (Applied Biosystems): OSM: 5′- GCTCACACAGAGGACGCTG-3′, 5′-GGAGCACGCGGTACTCTTTC-3′; GAPDH: 5′-TCGACAGTCAGCCGCATCTTCTTT-3′, 5′-ACCAAATCCGTTGACTCCGACCTT-3′. The PCR program steps were 95 °C for 10 min, 40 cycles of 95 °C for 15 s, and 60 °C for 1 min. The relative expression of mRNA for OSM was normalized by GAPDH and measured by using 2^−ΔΔCt^ method.

### 2.6. Western Blot

Exposure to GM-CSF led to the maximum phosphorylation of PI3K in 15 min and Akt/NF-κB in 30 min as determined in our previous study [17]. dHL-60 cells (1 × 10^7^ cells in 2 mL of medium) were seeded in 60 mm dish and pretreated with ST or vehicle for 1 h, and then stimulated with GM-CSF (5 ng/mL) for 15 min (PI3K) or 30 min of Akt or 30 min of NF-κB. An ice-cold cell lysis buffer (Sigma-Aldrich Co., Ltd., St. Louis, MO, USA) was used to lyse the harvested cells. Cell extracts were prepared with sampling buffer (Laemmli’s 2×, ELPISBIOTECH. INC., Daejeon, Republic of Korea) and heated at 95 °C for 5 min. Proteins were subjected to electrophoresis using 10–15% gel containing sodium dodecyl sulfate and transferred to nitrocellulose membranes (Amersham™, Chicago, IL, USA) as described previously [45,46,47]. PBST containing 5% bovine serum albumin (Sigma-Aldrich Co., Ltd., St. Louis, MO, USA) was used to block the membranes; afterwards, relevant primary antibodies (phosphorylated (p)-PI3K, Cat. No. 17366, 1:1000 dilution, Cell Signaling Technology, Danvers, MA, USA; PI3K, Cat. No. sc-423, 1:500 dilution, p-Akt, Cat. No. sc-514032, 1:500 dilution, Akt, Cat. No. sc-81434, 1:500 dilution, p-p65, Cat. No. sc-136548, 1:500 dilution, p65, Cat. No. sc-8008, 1:500 dilution, Actin, Cat. No. sc-8432, 1:500 dilution, and GAPDH, Cat. No. sc-32233, 1:500 dilution, Santa Cruz Biotechnology, Santa Cruz, CA, USA) were used. Peroxidase-conjugated secondary antibodies (m-IgGκ BP-HR, Cat. No. sc-516102, 1:5000 dilution, mouse anti-rabbit IgG-HRP, Cat. No. sc-2357, 1:5000 dilution, Santa Cruz Biotechnology, Santa Cruz, CA, USA) were added for incubation of the membranes for 1 h at room temperature after washing with PBST. Specific bands were detected by an enhanced chemiluminescence solution (DoGenBio Co., Seoul, Republic of Korea). Densitometric quantification was conducted with ImageJ program (version 1.53e, National health institute, Bethesda, MD, USA). Expression levels were normalized to Actin or GAPDH.

### 2.7. Immunofluorescence Staining

dHL-60 cells (2 × 10^6^ cells in 2 mL of medium) were seeded in a 60 mm dish and pretreated with ST or vehicle for 1 h, and then stimulated with GM-CSF (5 ng/mL) for 30 min. dHL-60 cells were fixed with 4% paraformaldehyde, permeabilized in 0.2% Triton X-100, and incubated with a blocking buffer (PBS containing 10% FBS) to reduce nonspecific binding as previously described [48,49]. The cells were incubated with the primary antibody (p-p65, Cat. No. sc-136548, 1:50 dilution, Santa Cruz Biotechnology, Santa Cruz, CA, USA), followed by incubation with Alexa Fluor^®^ conjugated secondary antibody (Alexa Fluor^®^ 647, Cat. No. ab150115, 1:1000 dilution, Abcam, Cambridge, MA, USA) at room temperature. For nuclear staining, 4′,6-diamidino-2-phenylindole (DAPI, Sigma-Aldrich Co., Ltd., St. Louis, MO, USA) was used. Samples were visualized under a confocal laser scanning microscope (Carl Zeiss, Oberkochen, Germany). Fluorescence intensity was measured by ZEN 2.3 (version 2.3.69.1000).

### 2.8. Statistical Analysis

All data are shown as the mean ± SD and analyzed using SPSS (version 29.0.2.0 (20)). The significance was evaluated using one-way analysis of variance (ANOVA) with Tukey’s post hoc test as well as an independent *t*-test. Comparisons across multiple groups (e.g., dose–response) used one-way analysis of variance (ANOVA) with Tukey’s post hoc test, while a comparison of two specific groups used an independent *t*-test. *p* < 0.05 indicated statistical significance.

## 3. Results

The maximal timepoints are as follows: OSM protein 4 h, OSM mRNA 30 min, PI3K 15 min, and Akt/NF-κB 30 min.

### 3.1. Decreased OSM Levels by ST in dHL-60 Cells

**Figure 2:**
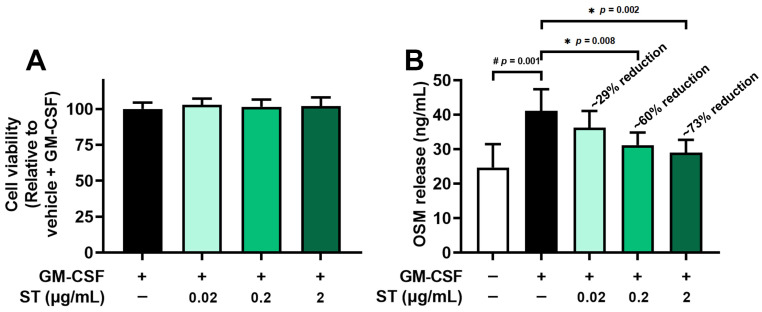
Effects of ST on OSM protein levels in dHL-60 cells. (**A**) Cell viability was assessed by an MTT assay. (**B**) OSM protein in medium was detected by ELISA method. Data are presented as the mean ± SD from the three separate experiments (n = 3). (# *p* < 0.05 vs. vehicle-treated and unstimulated group; * *p* < 0.05 vs. vehicle-treated and GM-CSF-stimulated group).

From the results of an MTT assay, a toxicity of ST to dHL-60 cells was not found in both differentiated (Figure 2A) and undifferentiated HL-60 cells (Figure S1). Pretreatment with ST (0.02, 0.2, and 2 μg/mL) for 1 h prior to incubation of dHL-60 cells with GM-CSF for 4 h was conducted to explore the effect of ST on OSM production. In line with a previous report [17], the incubation with GM-CSF resulted in increased OSM levels (i.e., 41.100 ± 6.316 for GM-CSF, Figure 2B). The increased levels were diminished by incubation with a wide range of doses of ST (0.02, 0.2, and 2 μg/mL) and the diminished values were as follows: 36.283 ± 4.819, 31.167 ± 3.688, and 28.983 ± 3.750. The value of unstimulated cells was 24.683 ± 6.812. The maximum inhibition rate of ST was about 73%, suggesting that ST partially reduces OSM release.

### 3.2. Decreased OSM mRNA by ST in dHL-60 Cells

**Figure 3:**
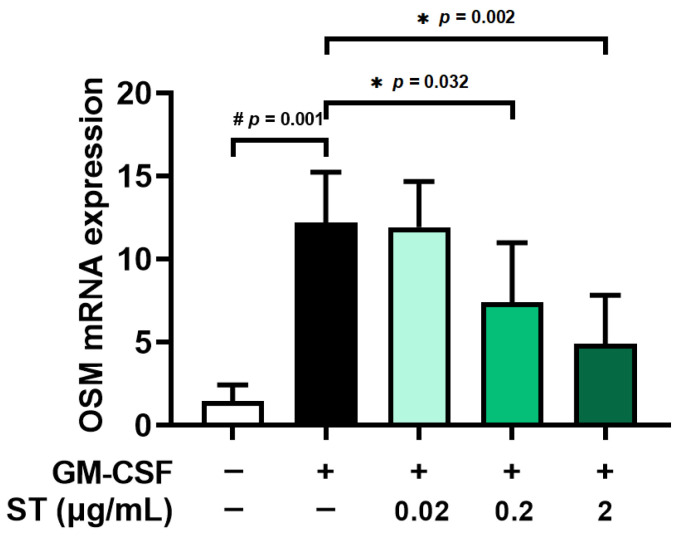
Effects of ST on OSM mRNA levels in dHL-60 cells. OSM mRNA levels were assessed by means of qRT-PCR method. Data are presented as the mean ± SD from the three separate experiments (n = 3). (# *p* < 0.05 vs. vehicle-treated and unstimulated group; * *p* < 0.05 vs. vehicle-treated and GM-CSF-stimulated group).

Pretreatment with ST (0.02, 0.2, and 2 μg/mL) for 1 h prior to incubation of dHL-60 cells with GM-CSF for 30 min was conducted to explore the effect of ST on mRNA expression of OSM. Similarly to the results of a previous report [17], the incubation with GM-CSF resulted in increased OSM mRNA levels (i.e., 12.187 ± 3.048 for GM-CSF, Figure 3). The elevated levels decreased through incubation with a variety of doses of ST (0.02, 0.2, and 2 μg/mL) and the decreased values are as follows: 11.926 ± 2.741, 7.403 ± 3.590, and 4.900 ± 2.931. The value of unstimulated cells was 1.436 ± 0.982. In the following studies (Western blot analysis and immunofluorescence staining), 2 μg/mL of ST was selected because it is non-toxic and represents a pharmacologically relevant concentration for probing the pathway.

### 3.3. A Decrease in Phosphorylated-PI3K by ST in dHL-60 Cells

**Figure 4:**
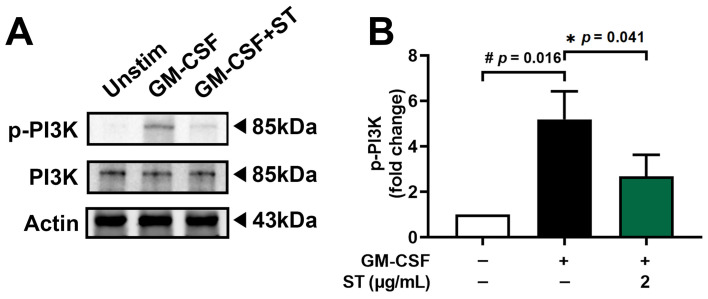
Effects of ST on phosphorylation of PI3K in dHL-60 cells. (**A**) Western blotting was performed for PI3K in dHL-60 cells. Unstim, vehicle-treated and unstimulated group; GM-CSF, vehicle-treated and GM-CSF-stimulated group; GM-CSF+ST, ST-treated and GM-CSF-stimulated group. (**B**) Blots were quantified by means of Image J program. Expression levels of p-PI3K were normalized to Actin. Data are presented as the mean ± SD from the three separate experiments. (# *p* < 0.05 vs. vehicle-treated and unstimulated group; * *p* < 0.05 vs. vehicle-treated and GM-CSF-stimulated group).

Pretreatment with 2 μg/mL (about 4.8 μM) of ST for 1 h prior to incubation of dHL-60 cells with GM-CSF for 15 min was conducted to find the regulatory mechanism of ST on OSM production. Phospho-PI3K increased 5.2-fold with GM-CSF and was reduced to 2.7-fold by ST (Figure 4B).

### 3.4. A Decrease in Phosphorylated-Akt by ST in dHL-60 Cells

**Figure 5:**
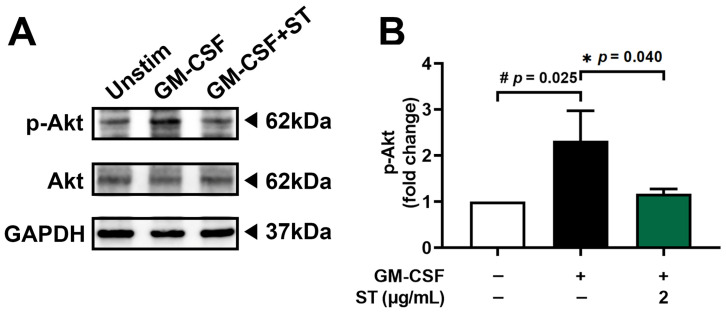
Effects of ST on phosphorylation of Akt in dHL-60 cells. (**A**) Western blotting was performed for Akt in dHL-60 cells. Unstim, vehicle-treated and unstimulated group; GM-CSF, vehicle-treated and GM-CSF-stimulated group; GM-CSF+ST, ST-treated and GM-CSF-stimulated group. (**B**) Blots were quantified by means of Image J program. Expression levels of p-Akt were normalized to GAPDH. Data are presented as the mean ± SD from the three separate experiments. (# *p* < 0.05 vs. vehicle-treated and unstimulated group; * *p* < 0.05 vs. vehicle-treated and GM-CSF-stimulated group).

Pretreatment with 2 μg/mL of ST for 1 h prior to incubation of dHL-60 cells with GM-CSF for 30 min was performed to find the modulatory mechanism of ST on OSM production. Phospho-Akt increased 2.3-fold with GM-CSF and was reduced to 1.2-fold by ST (Figure 5B).

### 3.5. A Decrease in Phosphorylated-NF-κB by ST in dHL-60 Cells

**Figure 6:**
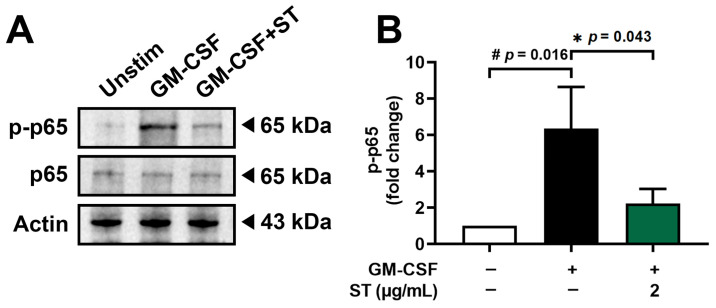
Effects of ST on phosphorylation of NF-κB in dHL-60 cells. (**A**) Western blotting was performed for NF-κB in dHL-60 cells. Unstim, vehicle-treated and unstimulated group; GM-CSF, vehicle-treated and GM-CSF-stimulated group; GM-CSF+ST, ST-treated and GM-CSF-stimulated group. (**B**) Blots were quantified by means of Image J program. Expression levels of p-p65 were normalized to Actin. Data are presented as the mean ± SD from the three separate experiments. (# *p* < 0.05 vs. vehicle-treated and unstimulated group; * *p* < 0.05 vs. vehicle-treated and GM-CSF-stimulated group).

Incubation of dHL-60 cells with GM-CSF for 30 min after pretreatment with 2 μg/mL of ST for 1 h was conducted to know the regulatory mechanism of ST on OSM production. Phospho-NF-κB (p-p65) increased 6.4-fold with GM-CSF and was reduced to 2.2-fold by ST (Figure 6B).

### 3.6. A Decrease in Phosphorylated-NF-κB Fluorescence Staining by ST in dHL-60 Cells

**Figure 7:**
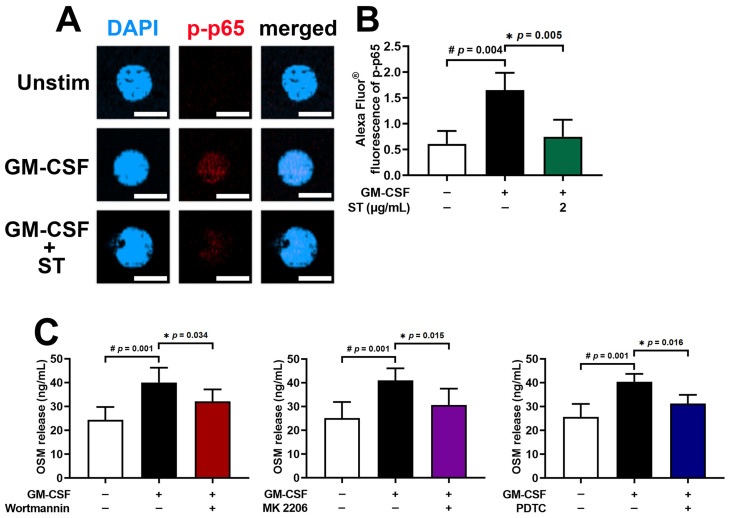
Effects of ST on phosphorylated NF-κB (p-p65) immunofluorescence staining in dHL-60 cells and confirmation of signal pathway. (**A**) p-p65 levels were assessed by immunofluorescence. Unstim, vehicle-treated and unstimulated group; GM-CSF, vehicle-treated and GM-CSF-stimulated group; GM-CSF+ST, ST-treated and GM-CSF-stimulated group. (**B**) The graphic shows the median of Alexa Fluor^®^ (red) fluorescence intensity. Fluorescence photograph of representative cell in each group is presented. Magnification ×400, scale bar: 10 μm. (**C**) dHL-60 cells were treated with each inhibitor (PI3K inhibitor-wortmannin-50 μM or Akt inhibitor-MK 2206-50 μM or NF-κB inhibitor-PDTC-100 μM). OSM protein in medium was detected by ELISA method. Data are presented as the mean ± SD from the three separate experiments (n = 3). # *p* < 0.05 vs. vehicle-treated and unstimulated group; * *p* < 0.05 vs. vehicle-treated and GM-CSF-stimulated group.

An immunofluorescence assessment for p-NF-κB (a critical and final stage of the PI3K/Akt/NF-κB pathways) was performed to verify the regulatory mechanism of ST in fluorescence staining. Stimulation with GM-CSF for 30 min was conducted in dHL-60 cells after preincubation with 2 μg/mL of ST for 1 h. Similarly to the results of a previous report [17], phosphorylated-NF-κB (p-p65) increased by exposure to GM-CSF (Figure 7A). As shown in Figure 7B, the increased phosphorylated-NF-κB (p-p65) decreased by incubation with ST. To demonstrate that the pathway (PI3K/Akt/NF-κB) is indeed responsible for OSM production in our model, PI3K inhibitor (wortmannin), Akt inhibitor (MK 2206), and NF-κB inhibitor (PDTC) were used. As shown in Figure 7C, OSM production was reduced by each inhibitor.

## 4. Discussion

Several studies have indicated that inflammatory respiratory disorders, inclusive of chronic rhinosinusitis and asthma, express up-regulated OSM values [16,50,51]. Exposure to GM-CSF resulted in up-regulated OSM mRNA levels [52]. Numerous studies have indicated that GM-CSF stimulation induces increased OSM secretion in separated human neutrophils [16,21,53,54]. In line with a previous report [17], increased mRNA and protein of OSM resulted from exposure to GM-CSF. In this study, addition of ST down-regulated the increased OSM mRNA and protein levels. It is thus possible to presuppose that ST may have potential implications for treating neutrophilic asthma. Mozaffarian et al. [55] have indicated that elevations of inflammatory infiltrate as well as inflammatory chemokines and cytokines are found in the lungs of OSM-treated mice. Another study reported that OSM injection leads to increased inflammatory reactions in the skin of mice and OSM treatment results in increased cytokine and chemokine expression in cells [56]. Pulmonary overexpression of OSM produced a robust induction of inflammatory reactions in mice [57]. The more severe the symptoms, the higher OSM levels in patients with asthma [51]. On the contrary, down-regulation of OSM (i.e., OSM neutralization antibody treatment and OSM eliminated mice) produced a reduction in inflammatory responses in a murine model [5]. We are able to presuppose that ST might be beneficial to avert inflammatory disorders via suppressing OSM.

The famous signaling cascade PI3K/Akt plays an important role in the regulation of inflammatory conditions [58,59,60,61]. Famous transcription factor NF-κB is essential in the regulation of inflammatory conditions. Su and colleagues [62] indicated that OSM release is controlled by the PI3K/Akt/NF-κB signaling cascade in MG-63 cells. Exposure to a PI3K inhibitor (LY294002) led to the down-regulation of diverse inflammatory factors (i.e., IL-1β, IL-6, and TNF-α) [63]. Additionally, the blockade of PI3K/Akt pathway led to the alleviation of joint disorders in a murine model [64]. A number of studies have indicated that exposure to diverse PI3K inhibitors, including wortmannin, IC87114, and LY294002 results in down-regulation of inflammatory conditions in the lungs of asthmatic mice [65,66]. Treatment with wortmannin (one of diverse PI3K inhibitors) led to down-regulation of OSM production in our previous report [17]. Exposure to deguelin (a PI3/Akt inhibitor) also decreased inflammatory conditions in the lungs of asthmatic mice [67]. El-Hashim and colleagues [68] reported that blocking NF-κB results in suppressed inflammatory conditions in the lungs of asthmatic mice. The results of the present study implied that incubation with ST inhibits activation of PI3K, Akt, and NF-κB. In addition, treatment with each inhibitor (PI3K inhibitor-wortmannin or Akt inhibitor-MK 2206 or NF-κB inhibitor-PDTC) resulted in down-regulation of OSM release. We thus assume that the PI3K/Akt/NF-κB signaling pathway may be involved in down-regulation of OSM by ST in dHL-60 cells. Khan and colleagues [69] reported that ST exerts anti-arthritic activity through down-regulation of NF-κB as well as MAPK signal pathways in rats. ST might affect not only the PI3K/Akt/NF-κB pathway but other parallel pathways (e.g., MAPK). In the present study, dHL-60 cells were chosen due to donor variability and short lifespan of primary neutrophils. Nevertheless, validation in primary neutrophils will be required for future work. In the present study, 2 μg/mL of ST was used. Batta et al. [70] reported that plasma concentrations of ST in rats increase from 0.1 μg/mL to 4 μg/mL when feeding them a diet containing 0.5% ST for 6 weeks. Thus, 2 μg/mL of ST would be an achievable plasma level from dietary intake or supplementation.

## 5. Conclusions

**Figure 8:**
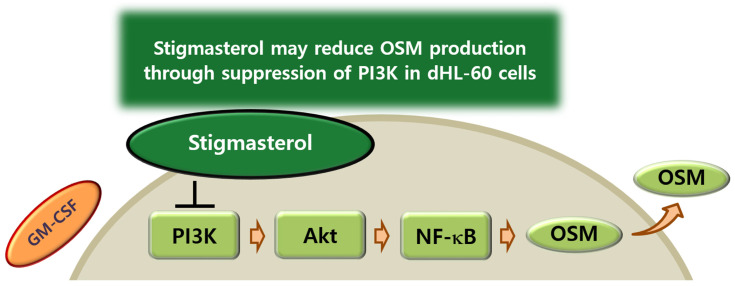
Proposed schematic diagram of OSM suppression by ST. ST may reduce OSM production through suppression of PI3K in dHL-60 cells.

In summary, the findings in this study showed that ST reduces OSM mRNA expression and production in dHL-60 cells. In addition, ST inhibited phosphorylation of PI3K, Akt, and NF-κB in dHL-60 cells. Lu and colleagues [71] reported that ST acts directly on PI3K through molecular docking simulation. We could thus presume that ST may reduce OSM production through suppression of PI3K in dHL-60 cells (Figure 8). Therefore, our data imply that ST may be beneficial to prevent inflammatory diseases. Even though our results showed that ST significantly inhibits OSM production, they were from a single cell line study. Therefore, future study will be needed to investigate in vivo pharmacokinetic/toxicology and efficacy studies.

## Data Availability

The original contributions presented in this study are included in the article/Supplementary Materials. Further inquiries can be directed to the corresponding author.

## Supplementary Materials

The following supporting information can be downloaded at https://www.mdpi.com/article/10.3390/biomedicines14010220/s1: Figure S1: Cell viability of ST in undifferentiated HL-60 cells. Figure S2: Uncropped photographs in immunofluorescence staining. Figure S3: Full-length blots and molecular weight markers.
