# Supplementary material for: Stigmasterol Decreases Oncostatin M Production Through Suppressing PI3K/Akt/NF-κB Signaling Processes in Neutrophil-like Differentiated HL-60 Cells
Source: Biomedicines. 2026 Jan 20;14(1):220. doi: 10.3390/biomedicines14010220 (PMC12839374; doi:10.3390/biomedicines14010220)
Supplement: Supplementary file 1 [file biomedicines-14-00220-s001.zip › biomedicines-4066912-supplementary.pdf]

biomedicines

Supplementary

Stigmasterol Decreases Oncostatin M Production Through Suppressing PI3K/Akt/NF-κB Signaling Processes in Neutrophil-like Differentiated HL-60 Cells

Na-Ra Han [1,2], Hi-Joon Park [3], Seong-Gyu Ko [2,4] and Phil-Dong Moon [5,*]

1 College of Korean Medicine, Kyung Hee University, Seoul 02447, Republic of Korea; nrhan@khu.ac.kr
2 Korean Medicine-Based Drug Repositioning Cancer Research Center, College of Korean Medicine, Kyung Hee University, Seoul 02447, Republic of Korea; epiko@khu.ac.kr
3 Department of Anatomy & Information Sciences, College of Korean Medicine, Kyung Hee University, Seoul 02447, Republic of Korea; acufind@khu.ac.kr
4 Department of Preventive Medicine, College of Korean Medicine, Kyung Hee University, Seoul 02447, Republic of Korea
5 Center for Converging Humanities, Kyung Hee University, Seoul 02447, Republic of Korea
* Correspondence: pdmoon@khu.ac.kr; Tel.: +82-2-961-0897

Results

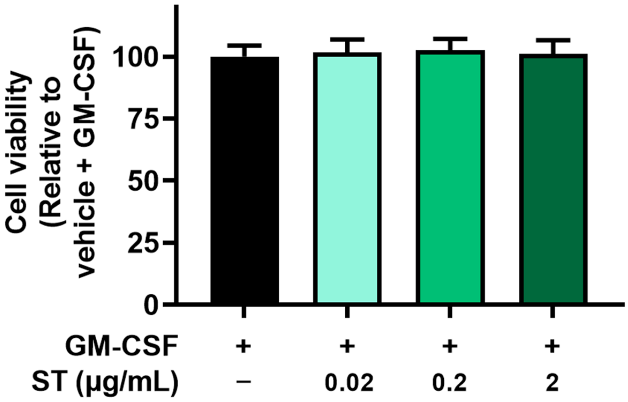

Figure S1. Cell viability of ST in undifferentiated HL-60 cells. Cell viability was assessed by an MTT assay. Data are presented as the mean ± SD from the three separate experiments (n = 3).

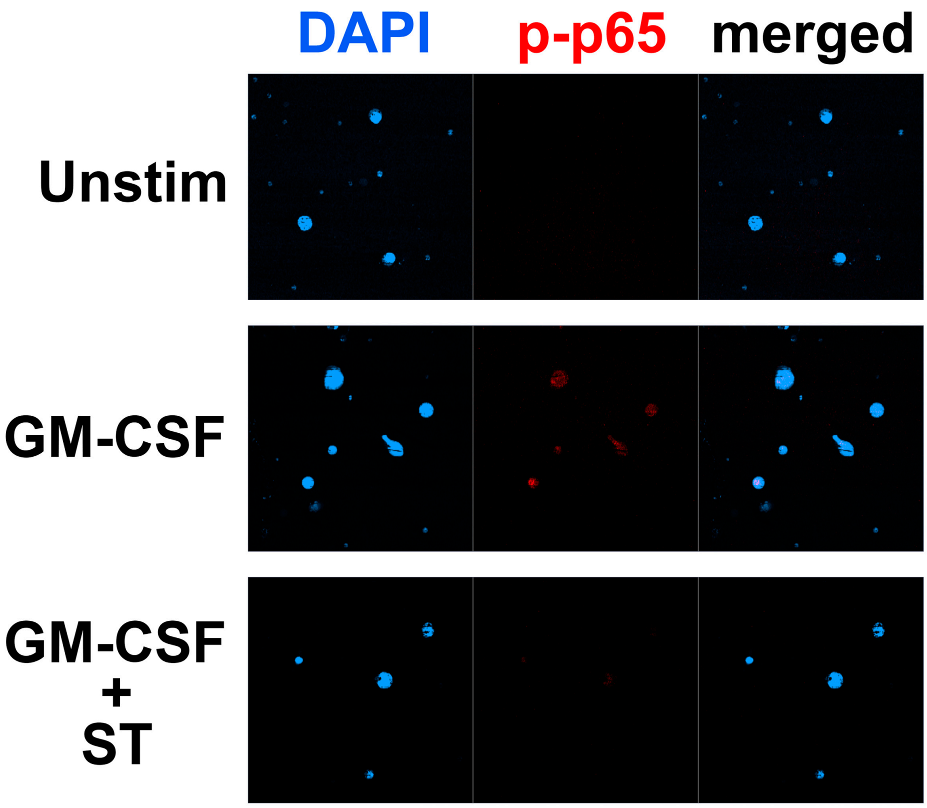

Figure S2. Uncropped photographs in immunofluorescence staining.

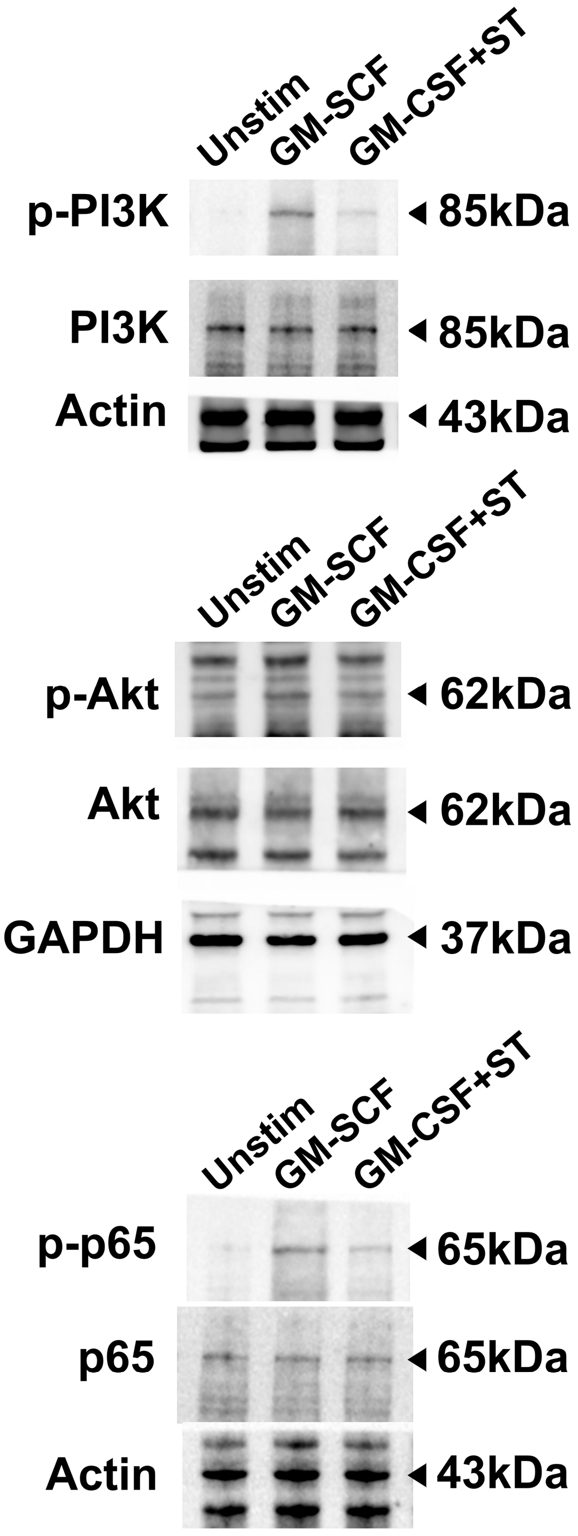

Figure S3. Full-length blots and molecular weight markers.
